# Histone acetyltransferase 1 upregulates androgen receptor expression to modulate CRPC cell resistance to enzalutamide

**DOI:** 10.1002/ctm2.495

**Published:** 2021-07-22

**Authors:** Zhe Hong, Zhendong Xiang, Pan Zhang, Qiang Wu, Chengdang Xu, Xinan Wang, Guowei Shi, Zongyuan Hong, Denglong Wu

**Affiliations:** ^1^ Department of Urology, Tongji Hospital, School of Medicine Tongji University Shanghai China; ^2^ Illinois Informatics Institute University of Illinois at Urbana‐Champaign Champaign Illinois USA; ^3^ Department of Urology, the Fifth People's Hospital of Shanghai Urology Research Center of Fudan University Shanghai China; ^4^ Laboratory of Quantitative Pharmacology Wannan Medical College Wuhu China

**Keywords:** androgen receptor, bromodomain containing protein 4, castration‐resistant prostate cancer, enzalutamide, histone acetyltransferase 1

## Abstract

Castration‐resistant prostate cancer (CRPC) is the latest stage of PCa, and there is almost no effective treatment available for the patients with CRPC when next‐generation androgen deprivation therapy drugs, such as enzalutamide (ENZ), fail. The androgen receptor (AR) plays key roles in PCa and CRPC progression and drug resistance. Histone acetyltransferase 1 (HAT1) has recently been reported to be highly expressed in some tumors, such as lung carcinoma. However, what relationship between the AR and HAT1, and whether or how HAT1 plays roles in CRPC progression and drug resistance remain elusive. In the present study, we found that HAT1 is highly expressed in PCa cells, and the overexpression of HAT1 is linked with CRPC cell proliferation. Moreover, the HAT1 expression is positively correlated with the expression of AR, including both AR‐FL (full‐length) and AR‐V7 (variant 7), which is mainly mediated by a bromodomain containing protein 4 (BRD4) ‐mediated pathway. Furthermore, knockdown of HAT1 can re‐sensitize the response of CRPC cells to ENZ treatment in cells and mouse models. In addition, ascorbate was observed to decrease AR expression through downregulation of HAT1 expression. Collectively, our findings reveal a novel AR signaling regulation pathway in PCa and CRPC and suggest that HAT1 serves as a critical oncoprotein and an ideal target for the treatment of ENZ resistance in CRPC patients.

## Graphical abstract

1. HAT1 is highly expressed in PCa cells and correlated with PCa and CRPC progression.
2. AR is transcriptionally upregulated by HAT1 through a BRD4‐madiated pathway.
3. Knockdown of HAT1 re‐sensitizes CRPC cells to enzalutamide treatment.

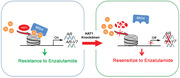

## 1 INTRODUCTION

Prostate cancer (PCa) remains the most frequently diagnosed malignant tumor in males.^1^ Although androgen deprivation therapy (ADT) is effective at the primary stage,^2^, ^3^ patients with PCa inevitably relapse into castration‐resistant prostate cancer (CRPC) after ADT treatment for 18–24 months.^4^, ^5^ It is reported that the median survival time of patients diagnosed with CRPC is usually less than 20 months.^6^, ^7^ However, an effective treatment for CRPC is still lacking. Therefore, the development of novel therapeutics for CRPC is urgently needed.

The androgen receptor (AR) is one of the most crucial factors that promote the progression of PCa and CRPC.^8^, ^9^ In prostate tissues, following binding with androgens, the AR and androgen complex translocate to the cell nucleus. Once in the nucleus, AR binds to androgen response elements upstream of target genes, leading to DNA transcription, promoting the abnormal proliferation of prostate epithelial cells and supporting oncogenesis.^9^, ^10^ Full‐length AR (AR‐FL) comprises NTD, DBD, and LBD three domains.^11^, ^12^ The LBD domain is the binding domain of enzalutamide (ENZ) on AR, and ENZ is currently the next‐generation of anti‐PCa drug.^13^ However, PCa cells develop AR variants (AR‐Vs) in CRPC,^14^ some of which lack the LBD domain and conferring resistance to ENZ treatments and leading to poor prognosis.^14^, ^15^ Among these AR‐Vs, AR‐V7 is the most notorious one that closely relates with ENZ resistance.^16^, ^17^

Histone acetyltransferase 1 (HAT1) is a type B histone acetyltransferase. In the process of chromatin assembly, HAT1 participates in the acetylation of newly synthesized histone H4^18^, ^19^ and is scarcely understood in cancer research.^20^, ^21^ It is reported that HAT1 drives histone H4 production by inducing chromatin acetylation and replication,^22^ and it is also over‐expressed in various types of tumors. Some researchers found that HAT1 may be an oncoprotein and promotes tumorigenesis.^23^, ^24^, ^25^, ^26^, ^27^ Additionally, HAT1 functions as a transcription factor in cancer cells and regulates the expression of various genes, including *Fas* and *Bcl2L12*,^24^, ^28^, ^29^ to promote lung and nasopharyngeal cancer cell proliferation, apoptosis, and metabolism. Moreover, a recent study reported that loss of HAT1 was involved in drug resistance in cancer treatment.^30^ However, what relationship between the AR (including AR‐V7) and HAT1, and whether or how HAT1 plays roles in CRPC progression and drug resistance remain elusive.

In this study, we found that HAT1 is highly expressed in PCa cells and has a positive correlation with PCa and CRPC progression. AR was upregulated by HAT1 through a bromodomain containing protein 4 (BRD4) ‐mediated pathway. Moreover, knockdown of HAT1 could re‐sensitize CRPC cells to ENZ treatment. Collectively, these results demonstrate that the abnormal expression of HAT1 promotes PCa and CRPC tumorigenesis by upregulating AR expression, and inhibition of HAT1 expression may shed new lights on CRPC treatment.

**HIGHLIGHTS**

- HAT1 is highly expressed in PCa cells and correlated with PCa and CRPC progression.
- AR is transcriptionally upregulated by HAT1 through a BRD4‐madiated pathway.
- Knockdown of HAT1 re‐sensitizes CRPC cells to enzalutamide treatment.

## 2 MATERIALS AND METHODS

### 2.1 Cell culture

LNCaP, C4‐2, and 22Rv1 human PCa cell lines and HEK‐293T human embryonic kidney cells were purchased from Chinese Academy of Science Cell Bank (Shanghai, China). 22Rv1, C4‐2, and LNCaP cells were cultured in RPMI‐1640 cell culture medium (Gibco). 293T cells were cultured in DMEM cell culture medium. Ten percent fetal bovine serum (Biological Industries) and 1% penicillin/streptomycin (HyClone) were supplemented to normal mediums. Steroid depleted medium was RPMI‐1640 cell culture one containing 10% charcoal/dextran stripped fetal bovine serum (CS‐FBS, ThermoFisher Scientific) and 1% streptomycin/penicillin (HyClone). Cells grow in a humid environment of 5% CO_2_ at 37°C. Cell lines used in this study were not contaminated by mycoplasma.

### 2.2 Antibodies and chemicals

HAT1 antibody (ab193097), KDM5C antibody (ab34718), and BRD4 antibody (ab128874) were purchased from Abcam. GAPDH antibody (5174S), AR antibody (5153S), H4K5ac antibody (8647S), Cleaved caspase 3 antibody (9661S), and Ki‐67 antibody (9027S) were purchased from Cell Signaling Technology. Ascorbate (S4245) and JQ1 (S7110) were purchased from Selleckchem company (Shanghai, China).

### 2.3 Western blotting

Cells were harvested and lysed in RIPA buffer with 1% phosphatase and protease inhibitors. Protein content was quantified by BCA protein quantification kits; the same amount (∼30 μg) of proteins was separated and transferred onto NC membranes (Millipore). After blocking membranes for 1 h at room temperature, the NC membranes were incubated with dilutions containing specific primary antibodies overnight at 4℃. Next day, the membrane was washed three times (10 min/each time) with 1 × TBST and incubated with HRP‐conjugated secondary antibodies for 1 h at room temperature. Finally, the membrane was washed three times (10 min/each time) again and visualized using the ECL exposure system (Thermo Fisher Scientific).

### 2.4 RNA isolation and qRT‐PCR

Total RNA was extracted and reversely transcribed into cDNA according to manufacturer's protocols/instruction. The Bio‐Rad CFX96 system (Takara) was utilized to conduct the real‐time PCR. Relative expression of target mRNA was determined by the 2^–ΔΔCt^ method (Ct, cycle threshold). GAPDH was used as the control. Primers for qRT‐PCR are provided in Table S1.

### 2.5 Cell proliferation assay

Cells were seeded into a 96‐well plate with a density of 1000 cells per well. After culturing for a period as the indicated, the cells were stained with 3‐(4, 5‐dimethylthiazol‐2‐yl)‐2, 5‐diphenyltetrazolium solution for 1 h and then dissolved with DMSO. Cell growth was detected by measuring OD value at 490 nm wavelength.

### 2.6 Wound healing assay

Cells were plated in 12‐well plates for a density of 70%. After completely adherent and growing to the density of 95%, cells were scratched two vertical wounds per well with 10 μl tips and starved in RPMI‐1640 with 0.5% FBS. The wound Images were captured to measure wound closure distances.

### 2.7 *In vivo* studies

Male NOD/SCID mice (Shanghai SLAC Laboratory Animal Company), 6 weeks old, 22‐24 g, were individually housed in Tongji hospital animal center with 60% ± 3% humidity, 22 ± 0.5°C temperature and automatically controlled light/dark cycle. Animals may freely access to food and water.

These mice were castrated and grouped. Cells were mixed with Matrigel (1: 1) and injected (s.c.) into the right flank of mice. The size of tumor in mice was detected by caliper externally every other day for consecutive 27 days after administration. The mice were administrated with ENZ (10 mg/kg/2 days, p.o.) at the time of the tumor volume reaching 50 mm^3^. Mice were sacrificed at due time, and tumors were collected and measured for further studies. The protocol of animal experiments was approved by the Animal Care Ethics Committee of Tongji Hospital, School of Medicine, Tongji University (Shanghai, China).

### 2.8 Lentiviral expression plasmids and virus production

The plasmids shControl, shHAT1, shBRD4, pTSin, and pTSin‐HA‐HAT1 were transfected with psPAX2 packaging plasmids and pMD2.G envelope plasmids into HEK‐293T cells using Lipofectamine 3000 (Invitrogen) transfection for 48 h to obtain lentivirus soups that were frozen at −80℃ for further study. Sequences of gene‐specific shRNAs are provided in Table S2.

### 2.9 Colony formation assay

Passaged cells were digested and re‐suspended in fresh medium. Cell densities were quantified and dispersed into 6‐cm cell culture plates with a density of 1000 cells/dish. On day 14, cells were harvested, and the medium was removed, and the cells were fixed using 1 ml of 4% paraformaldehyde for 20 min. Then removing 4% paraformaldehyde and the cells were stained using 1 ml of 0.1% crystal violet solution for 20 min. Next the crystal violet solution was removed, and 2 ml PBS was added, and it was allowed to stand for 5 min. Finally, removing PBS and washing the plates with pure water for three times and evaporating naturally for taking pictures and quantifying cell count.

### 2.10 The Cancer Genome Atlas, Oncomine, and GEPIA database and webtool

Patients’ clinical profiles and the expression of HAT1 in The Cancer Genome Atlas (TCGA) PCa cohort comprising 549 patients were obtained from http://ualcan.path.uab.edu/. The Oncomine data were obtained from https://www.oncomine.org, and the Gene Expression Profiling Interactive Analysis (GEPIA) data were obtained from http://gepia.cancer‐pku.cn/.

### 2.11 Human PCa and paracancerous prostate tissue samples

Parts of human PCa and paracancerous prostate tissue samples for detecting the protein level of HAT1 were obtained from the tissue specimen bank of the urological department of Tongji hospital. The experimental protocols were approved by the Ethics Committee of Tongji Hospital, School of Medicine, Tongji University (Shanghai, China).

### 2.12 H&E staining and immunohistochemistry staining

H&E and immunohistochemistry (IHC) analysis and IHC score calculation were as described previously.^31^ Anti‐HAT1 antibody (ab193097, Abcam, 1:1000) and anti‐AR antibody (5153S, Cell Signaling Technology, 1:500) were used to determine the expressions of HAT1 and AR in cancer and paracancerous tissues. Anti‐Ki‐67 antibody (9027S, Cell Signaling Technology, 1:400) and anti‐cleaved caspase 3 antibody (9661S, Cell Signaling Technology, 1:200) were used to detect the expressions of Ki‐67 and cleaved caspase 3 in mouse xenograft tumors. Two experienced pathologists (unaware of tissue information) independently evaluated and scored the intensity of IHC in FFPE samples.

### 2.13 Chromatin immunoprecipitation quantitative PCR

Chromatin immunoprecipitation (ChIP) assay was performed by applying the ChIP Kit according to manufacturer's instruction. DNA fragments were purified and analyzed by quantitative PCR to measure DNA binding intensity utilizing the PCR Reagents and Kit (Abcam, ab270816) according to manufacturer's instruction. Table S3 shows the primers for ChIP‐qPCR

### 2.14 Analysis of statistics

Data were from three independent experiments and presented as the mean ± standard deviation (SD) and analyzed using SPSS 19.0 software (SPSS Inc., USA). The statistical methods include ANOVA, Student's *t*‐test, Pearson's and Spearman's correlation analysis for the relationship between variables. *p* < 0.05 shows statistical significance.

## 3 RESULTS

### 3.1 HAT1 is upregulated in patients with PCa

**FIGURE 1:**
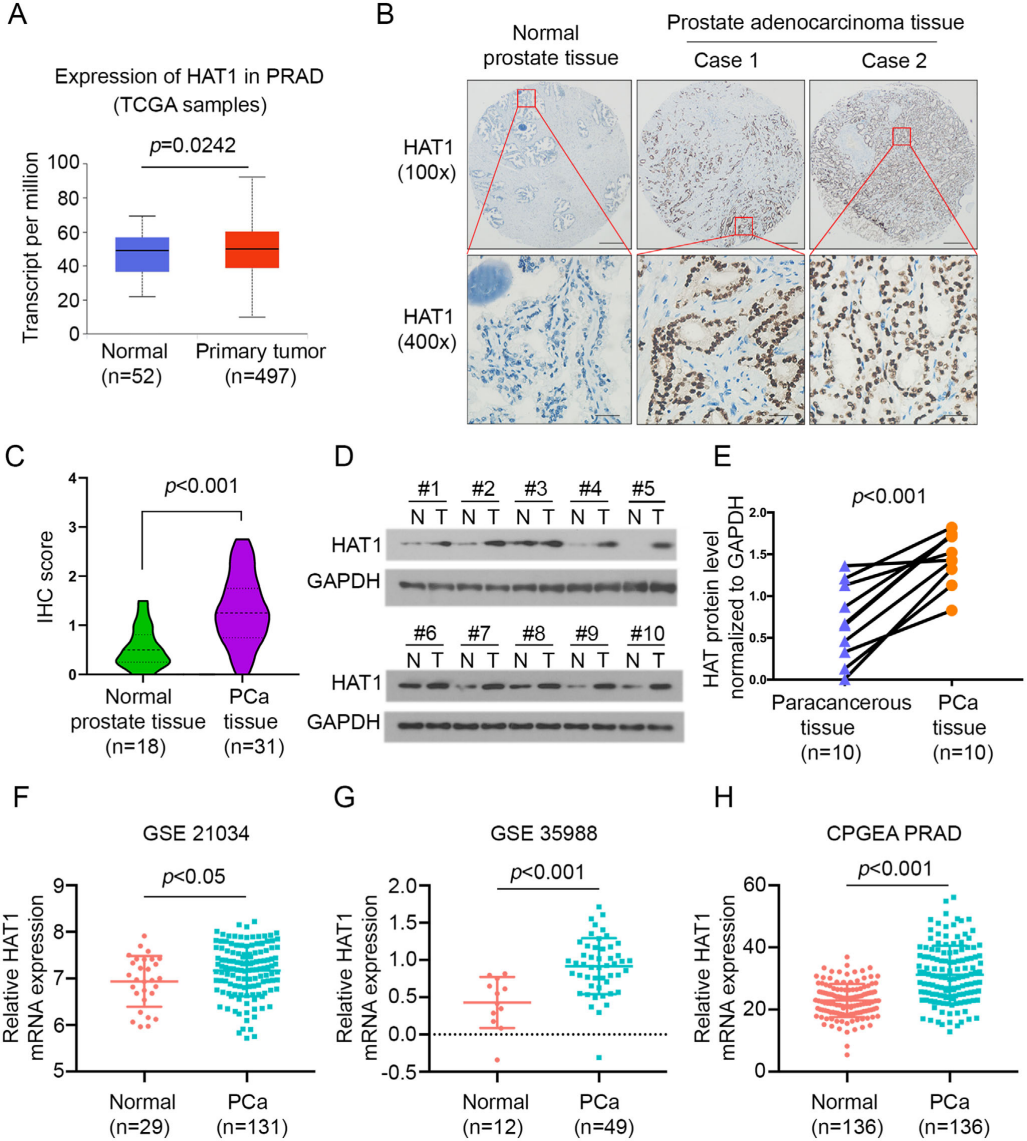
HAT1 is upregulated in PCa patients. (A) The expressions of HAT1 in normal prostate tissues and primary PCa tissues revealed by TCGA database. (B) Representative images for IHC analysis of HAT1 expression in PCa TMA specimens (normal prostate tissues, *n* = 18; PCa tissues, *n* = 31) in prostate tissue sections. Scale bars are shown as indicated. (C) Violin plot of HAT1 expressions in prostate tissue sections determined by IHC scores (normal prostate tissues, *n* = 18; PCa tissues, *n* = 31). (D and E) Western blot analysis of HAT1 expression in 10 paired PCa tissues (T) and the matched paracancerous non‐tumor tissues (N) from the same patient. GAPDH serves as a loading control, and the protein levels of HAT1 in D were quantified with ImageJ software. (F‐H) The transcriptional level of HAT1 in three independent studies revealed from CPGEA and silico datasets. Data were presented as the mean ± SD. Error bars indicate SD. The *p* values were analyzed using Student's *t*‐test and are shown as indicated

To explore the HAT1 expression level in PCa, the HAT1 expression in normal prostate tissues and primary prostate tumor tissues in TCGA database was assessed. We found that HAT1 expression in PCa tissues was higher than that in non‐tumor prostate tissues (Figure 1A). We then sought to determine HAT1 protein levels in PCa specimens using tissue microarray based on the prostate tissues (normal prostate specimens: *n* = 18; PCa specimens: *n* = 31). The scores of IHC staining were measured by considering both the percentage of positive staining cells and the staining intensity. The results confirmed the finding that the expression of HAT1 was significantly higher in PCa specimens than in normal prostate tissues (Figures 1B and 1C). Additionally, we detected the HAT1 protein level in PCa tissues and paracancerous prostate tissues and found that HAT1 was upregulated in PCa tissues (Figures 1D and 1E). We further compared the HAT1 mRNA expression between PCa samples and normal tissues based on RNA‐sequence data from Chinese Prostate Cancer Genome and Epigenome Atlas. Similarly, the mRNA expression of HAT1 was higher in PCa tissues than that in healthy tissues (Figure 1F). HAT1 mRNA expression was also higher in PCa primary tumors than that in the normal tissues in GSE21034 (Figure 1G) and GSE35988 (Figure 1H). Collectively, these data indicate that HAT1 is highly expressed in PCa compared with normal prostate.

### 3.2 HAT1 accelerates PCa and CRPC cell proliferation *in vitro* and *in vivo*

**FIGURE 2:**
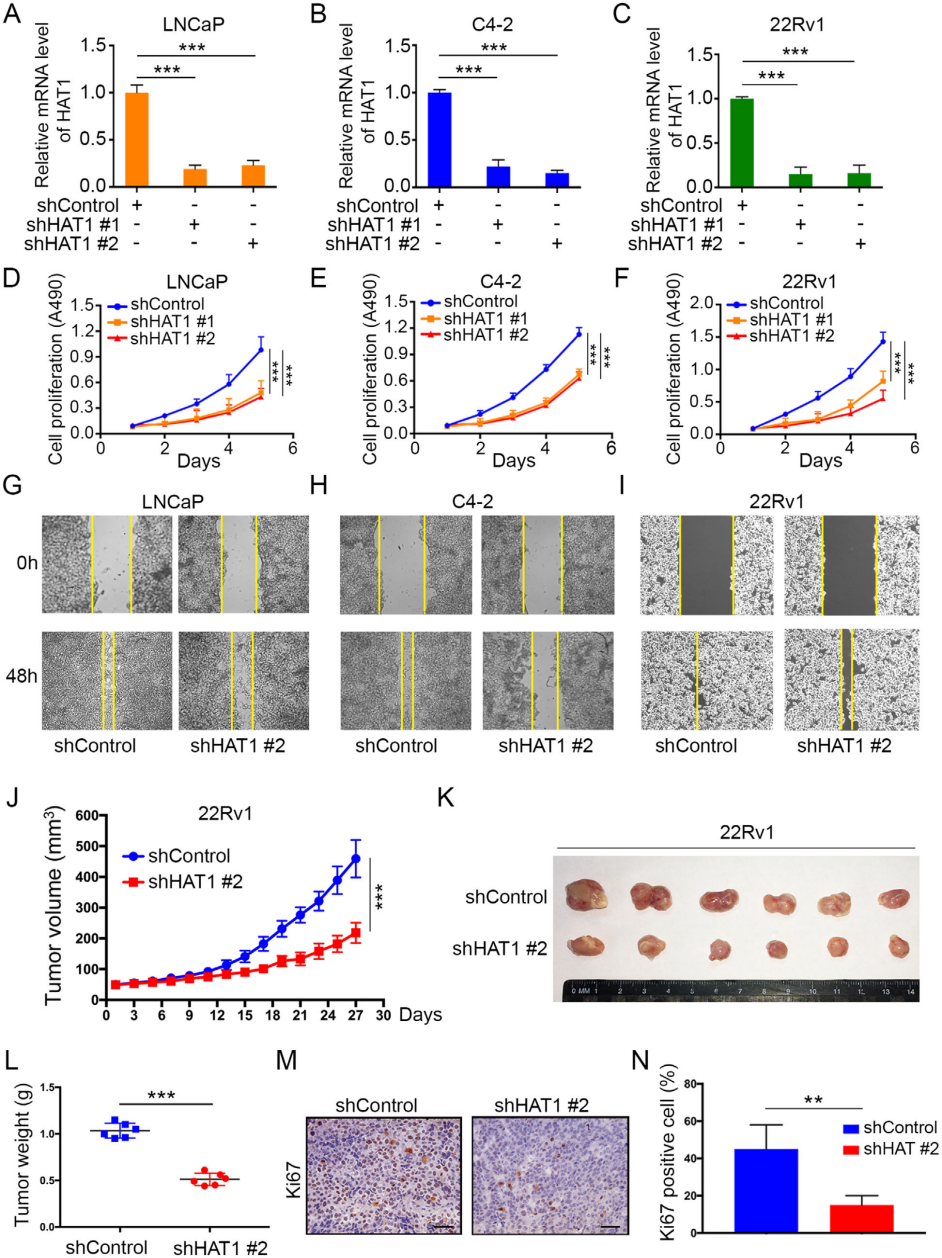
HAT1 accelerates PCa and CRPC cell proliferation *in vitro* and *in vivo*. (A‐F) LNCaP, C4‐2, and 22Rv1 cells were infected with lentivirus vectors expressing control or HAT1‐specific shRNAs for 48 h and selected with puromycin (10 μg/ml) for another 72 h. The cells then underwent qRT‐PCR analysis (A‐C) and MTS assays (D‐F). (G‐I) Wound‐healing assay of LNCaP, C4‐2 and 22Rv1 cells after infection with lentivirus vectors expressing control or HAT1‐specific shRNAs for 48 h and selected with puromycin (10 μg/ml) for another 72 h. (J‐L) 22Rv1 cells were infected with lentivirus vectors expressing control or HAT1‐specific shRNAs and injected subcutaneously (s.c.) into the right dorsal flanks of NOD‐SCID mice. Tumor growth was measured every other day for 27 days. Tumor volume at each time point was documented (J), tumors in each group at day 27 were harvested and photographed (K), and the tumor weights were also measured (L). (M and N) IHC staining of the Ki‐67 in tumor xenografts was conducted (M), and the staining scores were quantified (N). Data were presented as the mean ± SD of three independent experiments. Error bars indicate SD. The *p* values were analyzed using ANOVA (A‐F) and Student's *t*‐tests (J, L, and N). **p < 0.01, ***p < 0.001

Since that HAT1 is overexpressed in PCa tissues, we hypothesized that HAT1 may be involved in the oncogenesis of PCa. To verify this hypothesis, we knocked down HAT1 in three PCa cell lines, LNCaP, C4‐2, and 22Rv1 cells, with a pair of specific lenti‐virus short hairpin RNAs (shRNA) (Figures 2A–2C). Among these cell lines, LNCaP is considered a hormone‐sensitive PC (HSPC) cell line,^32^ C4‐2 is derived from LNCaP but with an increased metastatic potential to lymph nodes and bone,^33^ and 22Rv1 cell is derived from CWR xenograft with mouse under castrasted conditions.^34^ Both C4‐2 and 22Rv1 are cell lines with CRPC that show independent growth without androgens.^35^, ^36^ We found that with HAT1 knockdown, cell growth of all three cell lines was impeded as shown in an MTS assay (Figures 2D–2F). Additionally, results of a cell scratch assay indicated that the cell migration ability decreased with HAT1 knockdown (Figures 2G–2I and S2A). Consistently, when HAT1 was overexpressed in these three cell lines by transfecting HA‐tagged HAT1 plasmids (Figures S1A‐S1C), we found that the cell proliferation increased (Figures S1D‐S1F). The cell ‐proliferation promoting effect of HAT1 was previously described in colon cancer and lung cancer.^23^, ^24^

To investigate the function of HAT1 in PCa progression *in vivo*, we subcutaneously injected 22Rv1 cells with HAT1 knockdown into NOD/SCID mice for xenograft assays. Consistent with the findings from the *in vitro* assays, we found that HAT1 knockdown reduced the growth of 22Rv1 xenografts in mice (Figures 2J–2L). The xenografts then underwent IHC staining to detect the expression of Ki‐67, a commonly used marker for cell proliferation. We found that Ki‐67 staining is weaker in the HAT1 knockdown group than that in the control group (Figures 2M and 2N). We then constructed a new 22Rv1 cell line with stable HAT1 overexpression by infecting cells with *pTsin*‐HA‐HAT1 lentivirus. We performed the mouse xenograft assays by injecting (s.c.) these cells into the NOD/SCID mice. The results showed that HAT1 overexpression promotes PCa tumor growth *in vivo* (Figures S1G‐S1I). Collectively, all these results strongly indicate that HAT1 serves as an oncoprotein in PCa and CRPC and promotes cancer cell proliferation both *in vitro* and *in vivo*.

### 3.3 HAT1 regulates AR expression transcriptionally in PCa cells

**FIGURE 3:**
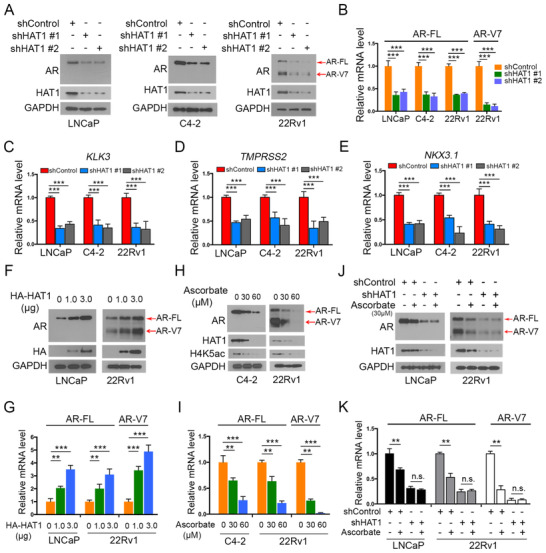
HAT1 regulates AR expression transcriptionally in PCa cells. (A) LNCaP, C4‐2, and 22Rv1 cells were infected with lentivirus vectors expressing control or HAT1‐specific shRNAs for 48 h and selected with puromycin (10 μg/ml) for another 72 h. The cells were then harvested for western blot analysis. (B‐E) LNCaP, C4‐2, and 22Rv1 cells were infected with lentivirus vectors expressing control or HAT1‐specific shRNAs for 48 h and selected with puromycin (10 μg/ml) for another 72 h. The cells were then harvested for qRT‐PCR analysis. (F and G) LNCaP and 22Rv1 cells were transfected with the indicated volume of plasmids, then cells were harvested for western blot analysis (F) and qRT‐PCR analysis (G) 24 h post‐transfection. (H and I) C4‐2 and 22Rv1 cells were treated with the indicated concentrations of ascorbate for 24 h, and cells were harvested for western blot analysis (H) and qRT‐PCR analysis (I). (J and K) LNCaP and 22Rv1 cells were infected with lentivirus vectors expressing control or HAT1‐specific shRNAs for 48 h, selected with puromycin (10 μg/ml) for another 72 h, and then treated with or without ascorbate for 24 h. Cells were harvested for western blot analysis (J) and qRT‐PCR analysis (K). Data were presented as the mean ± SD of three independent experiments. Error bars indicate SD. The *p* values were analyzed using ANOVA. ***p < 0.001

As the above data showed that HAT1 promotes PCa cell proliferation in both HSPC and CRPC cells, there are more biological functions of HAT1 to explore. We aimed to study the mechanisms by which HAT1 promotes PCa progression. AR is a well‐known oncoprotein and one of the most crucial oncogenic factors that promotes the cell proliferation of both HSPC and CRPC.^8^ Considering that the function of HAT1 in PCa is not well understood, we explored whether there is a correlation between HAT1 and AR. Surprisingly, we found that after knocking down HAT1, the protein level of AR decreased in LNCaP, C4‐2 and 22Rv1 cells (Figure 3A). Notably, we found that in 22Rv1 cells, the AR‐FL and AR‐V7 were dramatically decreased, the quantified data are shown in Figure S2E. Then, we detected the mRNA level of AR‐FL in three cell lines and AR‐V7 level in 22Rv1 cells. AR‐V7, as a member of AR‐Vs, is considered the most functional AR‐Vs in PCa progression and one of the most important drivers for CRPC progression.^16^ Our data showed that HAT1 knockdown decreased the mRNA level of AR‐FL in LNCaP, C4‐2 and 22Rv1 cells, and AR‐V7 in 22Rv1 cells (Figure 3B); also we observed that the decreased level of AR‐V7 was more dramatic than that of AR‐FL in mRNA and protein levels (Figures 3A and 3B). These data indicated that HAT1 regulates AR expression at the transcriptional level. Because the AR mRNA and protein expression levels decreased after HAT1 knockdown, we further detected the AR downstream target genes to see whether HAT1 could influence the AR signaling pathway. We observed that with HAT1 knockdown, AR target genes *KLK3*, *NKX3.1*, and *TMPRSS2* decreased in three independent cell lines (Figures 3C–3E). Alternatively, when we overexpressed HAT1 in LNCaP and 22Rv1 cells by ectopically transfecting HA‐HAT1 plasmids, we observed a dramatic increase of AR expression at both protein and mRNA levels (Figures 3F and 3G), and the AR‐V7 enjoyed a greater rising with HAT1 transfection. We also detected the AR downstream target gene expression with HA‐HAT1 transfection and found a significant increased *KLK3*, *TMPRSS2*, and *NKX3.1* expressions after transfection (Figure S2B).These results confirmed the finding that HAT1 positively regulates AR expression transcriptionally but have varying degrees for AR‐FL and AR‐V7. We also knocked down HAT1 and AR in 22Rv1 cells and found that the oncogenic effect of HAT1 on cell growth was in AR‐dependent pathway (Figure S2D).

Ascorbate (vitamin C) can decrease HAT1 expression epigenetically by TET‐mediated DNA hydroxymethylation.^37^ We treated 22Rv1 and C4‐2 cells with increasing concentrations of ascorbate. As expected, the expression of HAT1 and AR both decreased at protein and mRNA levels (Figures 3H and 3I). To further elucidate if the negative effect of ascorbate on AR is through HAT1, we detected AR expression in HAT1 knockdown and control cells with or without ascorbate treatments. The data showed that the expression of AR was downregulated after treatment with ascorbate, but the effect was abolished after HAT1 knockdown in 22Rv1 and LNCaP cells (Figure 3J); the quantified data are shown in Figure S2E. This trend was also observed at the mRNA level (Figure 3K), implying that HAT1 plays a pivotal role in the process of AR decrease after ascorbate treatment. Together, these data demonstrate that HAT1 upregulates both the AR‐FL and AR‐V7 expressions in PCa and CRPC cells at the transcriptional level.

### 3.4 AR expression in patient specimens is positively correlated with HAT1

**FIGURE 4:**
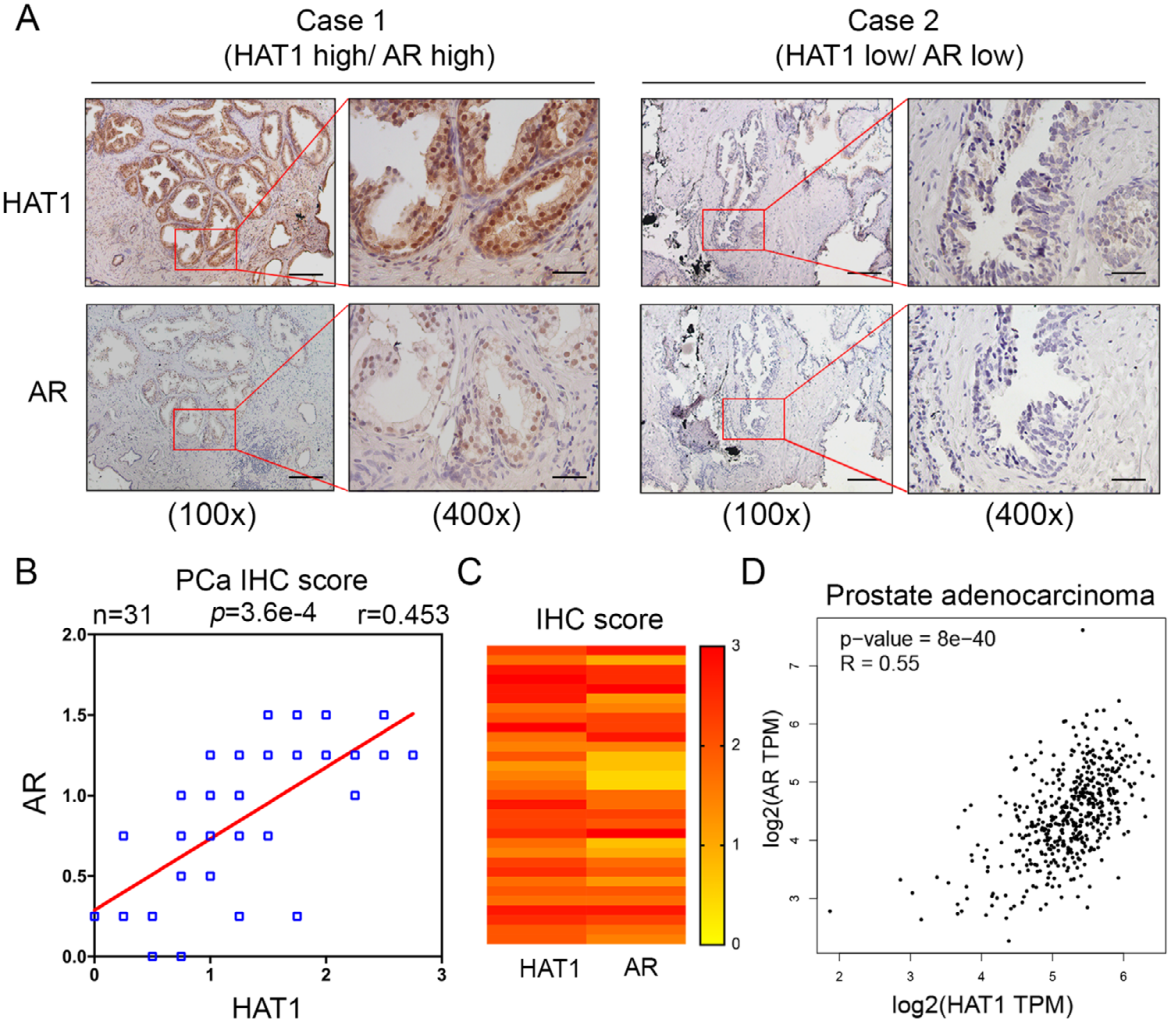
AR expression in patient specimens is positively correlated with HAT1. (A) Images for IHC staining of HAT1 and AR in PCa tissue sections. Scale bars are shown as indicated. (B and C) The correlation analysis (B) (*n* = 31) and heatmap analysis (C) of HAT1 and AR from the IHC staining scores in (A) are shown. (D) Correlation of HAT1 and AR mRNA expression in human PCa samples was determined by the GEPIA webtool. The co‐efficiency *r* or *R* and *p* values were calculated using Pearson's correlation (B) and Spearman's correlation (D) analyses, respectively and are shown as indicated

To further investigate the relationship between AR and HAT1 in PCa patients, 31 cases of PCa tissue samples were subjected to IHC analysis to test the expression of HAT1 and AR. The staining index of IHC was determined by measuring both the staining intensity and the percentage of positively stained cells, and images of the staining of HAT1 and AR are shown in Figure 4A. We found that the expression of HAT1 in patient samples is positively correlated with AR expression (Pearson's correlation coefficient *r* = 0.453, *p* < 0.001; Figures 4B and 4C). Then, we searched an online database using the GEPIA webtool, and intriguingly, the results showed that the mRNA level of AR is positively correlated with HAT1 in PCa patients (Spearman's correlation coefficient *R* = 0.55, *p *< 0.001; Figure 4D), consistent with our finding. These results suggest that in PCa patient specimens, AR expression is positively correlated with HAT1.

### 3.5 HAT1 knockdown re‐sensitizes CRPC cells to ENZ treatment

**FIGURE 5:**
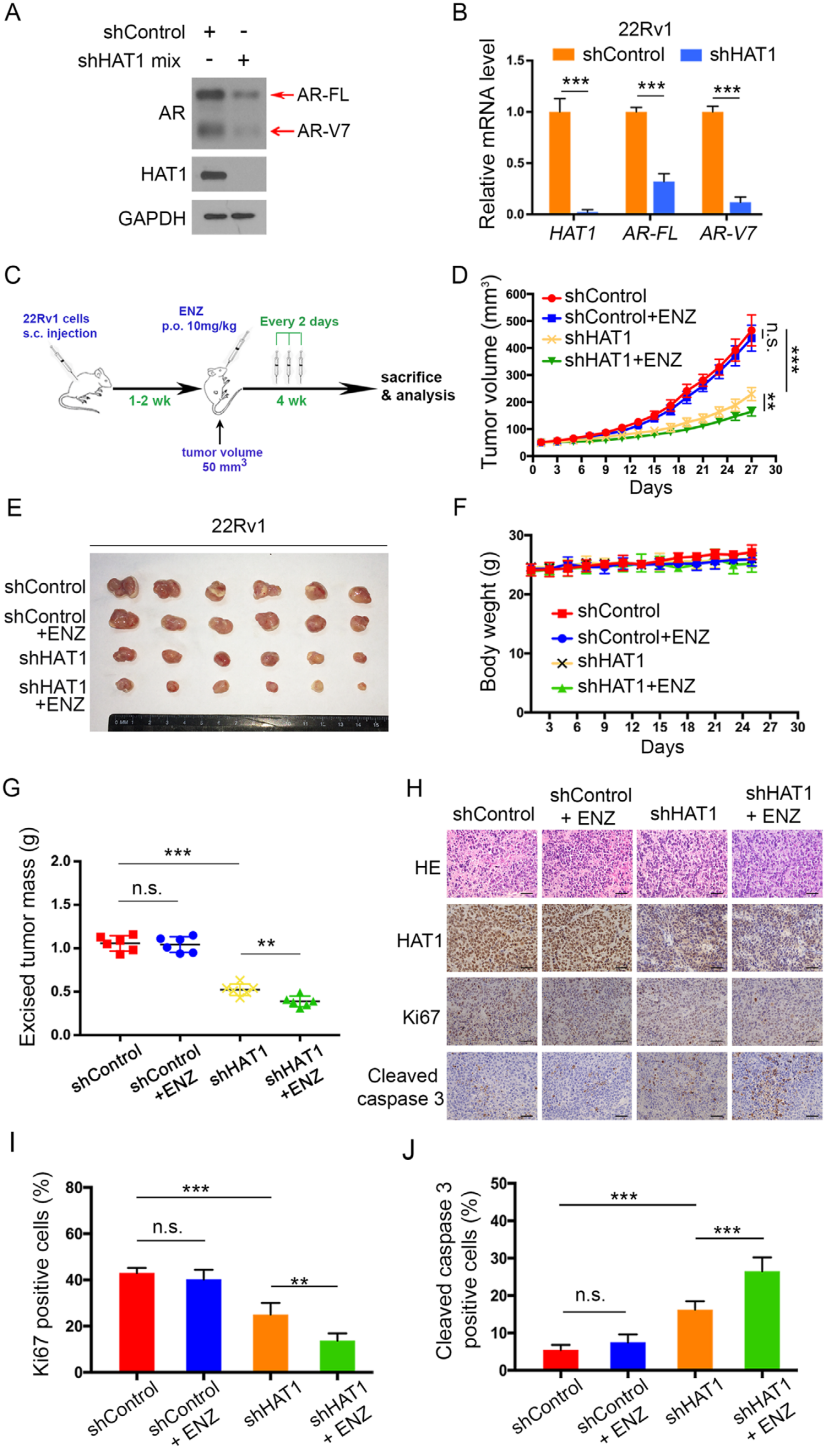
HAT1 knockdown re‐sensitizes CRPC cells to enzalutamide treatment. (A and B) 22Rv1 cells were infected with lentivirus vectors expressing control or mixed HAT1‐specific shRNAs for 48 h and selected with puromycin (10 μg/ml) for another 72 h. The cells were then harvested for western blot analysis (A) and qRT‐PCR analysis (B). (C) The schematic diagram demonstrating the drug treatment method for mice bearing subcutaneous tumors. (D‐G) 22Rv1 cells from (A) were mixed with Matrigel and injected subcutaneously (s.c.) into the right dorsal flanks of NOD/SCID mice and then treated with enzalutamide (10 mg/kg, p.o.) every 2 days when the average size of tumors reached 50 mm^3^. The growth curves of tumors were measured (D), and the tumors were harvested at day 27 and photographed (E). The mice (F) and tumors (G) were weight. (H) H&E staining and IHC staining for HAT1, Ki‐67 and cleaved caspase 3 were performed. Representative images were taken from each group. Scale bars are indicated in the images, scale bar = 50 μm. (I and J) Ki‐67 (I) and cleaved caspase 3 (J) positive cells in tissue sections obtained from (H) were quantified. The number of positive cells from at least five fields was counted and analyzed (I and J). Data were presented as the mean ± SD of three independent experiments (A‐G). Error bars indicate SD. The *p* values were analyzed using Student's *t*‐test. **p < 0.01, ***p < 0.001

The above results showed that HAT1 positively regulated AR expression in CRPC cells and may have potent effect on AR‐V7 (Figure 3). To define whether there is a therapeutic significance of HAT1 on PCa, especially CRPC, treatments, we knocked down HAT1 in 22Rv1 cells. As expected, knockdown of HAT1 resulted in a decrease of the AR‐FL and AR‐V7 expressions and consistently more serious on AR‐V7 (Figures 5A and 5B). Since 22Rv1 cells are resistance to ENZ treatment due to the high expression of AR‐V7,^15^ and HAT1 knockdown can reduce the AR‐FL and AR‐V7expressions, we aimed to test whether HAT1 can improve ENZ efficacy as a CRPC treatment. To test this hypothesis, we performed *in vitro* assays with 22Rv1 cells cultured in a hormone‐depleted environment (RPMI‐1640‐CS‐FBS), and the result showed that ENZ treatment exerted a significant impact on shHAT1 group, while such treatment had a less effect on shControl group, which indicated that HAT1 knockdown re‐sensitizes cells to ENZ (Figures S3A‐S3C). Also, the cell cycle distribution revealed a similar trend that the cells were mainly distributed in G1 phase with ENZ treatment on shHAT1 cells (Figure S4D). On the contrary, when we overexpressed HAT1 in C4‐2 cells, the cells showed an obvious resistance to ENZ treatment with both an increased IC50 and a reduced inhibitory rate on cell growth (Figures S3D‐S3F). Furthermore, 22Rv1 cells with HAT1 knockdown were injected (s.c.) into the right flank of NOD‐SCID male animals that were castrated before the injection. The mice bearing 22Rv1 tumors were then treated with or without ENZ orally when the tumor sizes reached 50 mm^3^ (Figure 5C). As demonstrated in Figures 5D–5G, tumor growth is markedly decreased in shHAT1 cells compared with shControl cells, consistent with the results from the *in vitro* assays (Figures S3A‐S3C). As reported by other groups,^38^ 22Rv1 cells showed no response to ENZ treatment, and we observed the same trend (Figure 5F). Strikingly, we found that in the shHAT1 group, when combined with the treatment of ENZ, tumor growth was significantly decreased and showed the slowest growth among the four groups (Figure 5D), and the tumor mass excised is the lowest among these four groups (Figure 5G). We further detected the IHC staining of HAT1, Ki‐67 and cleaved caspase 3 in tumor samples embedded with paraffin and found that the shHAT1 with ENZ treatment group induced increased apoptosis compared to the other groups (Figure 5H). Quantitative data of Ki‐67 and cleaved caspase 3 are shown in Figures 5I and 5J. As an AR positive cell line, 22Rv1 cells express a relatively high level of AR. We detected the HAT1 expression in multiple PCa cell lines and found a distinct expression in DU145 cells, an AR negative cell line (Figure S4A). When knockdown of HAT1 in DU145 cells, ENZ failed in retarding cell growth (Figures S4B and S4C). Together, these data suggest that HAT1 inhibition can decrease PCa growth substantially and notably re‐sensitize the CRPC cells to ENZ treatment.

### 3.6 HAT1 increases AR expression through BRD4 in PCa cells

**FIGURE 6:**
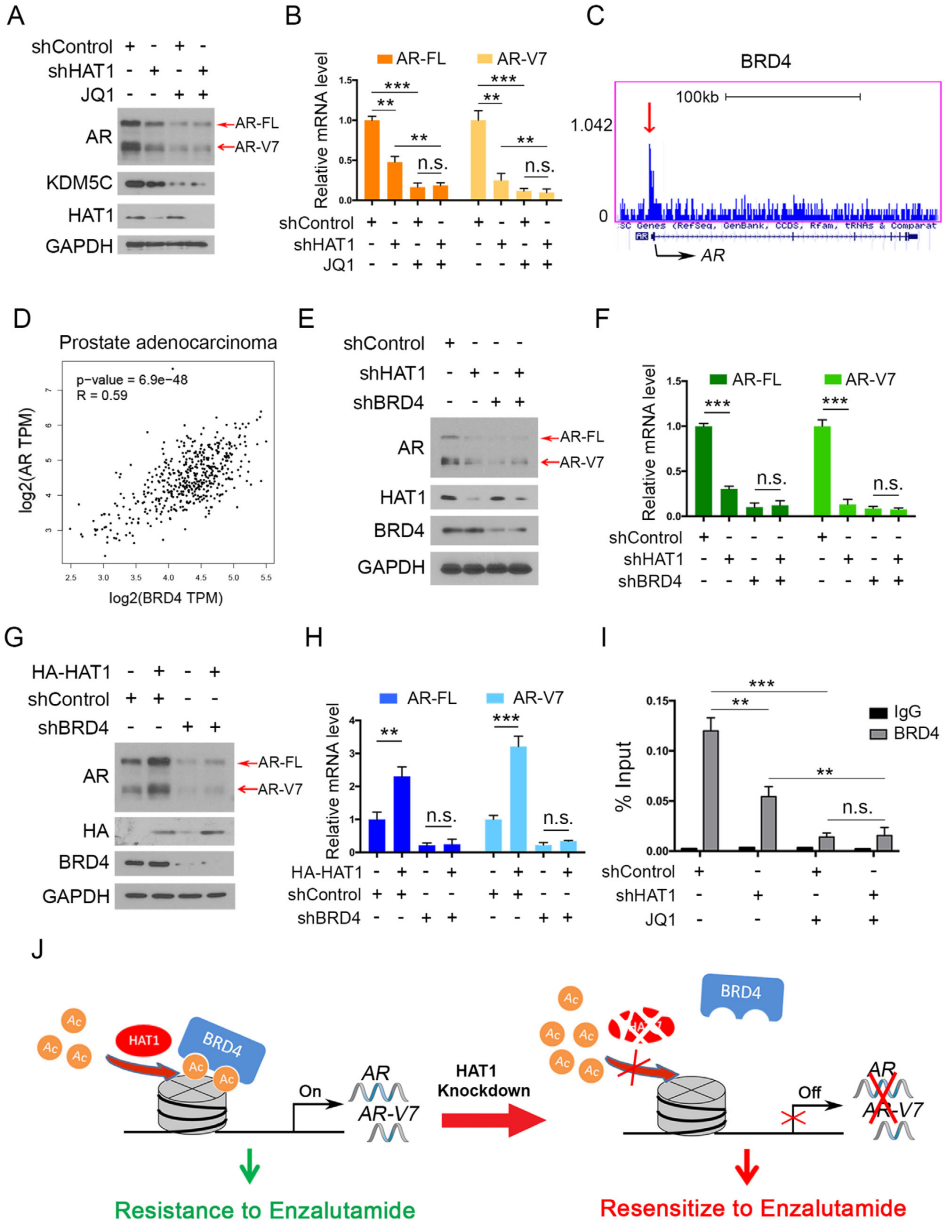
HAT1 increases AR expression through BRD4 in PCa cells. (A and B) 22Rv1 cells were infected with lentivirus vectors expressing control or HAT1‐specific shRNAs for 48 h and selected with puromycin (10 μg/ml) for another 72 h. The cells were then treated with or without JQ1 (3 μM) for 24 h and harvested for western blot analysis (A) and qRT‐PCR analysis (B). (C) The condition of binding BRD4 to the AR promoter in UCSC Genome‐Browser ChIP‐sequence data. Red arrow denotes the binding peak. (D) Correlation of BRD4 and AR mRNA expression in human PCa samples was determined by the GEPIA webtool. (E and F) 22Rv1 cells were infected with lentivirus vectors expressing control or HAT1‐specific shRNAs or BRD4‐specific shRNAs for 48 h and selected with puromycin (10 μg/ml) for another 72 h. The cells were then harvested for western blot analysis (E) and qRT‐PCR analysis (F). (G and H) 22Rv1 cells were infected with lentivirus vectors expressing control or BRD4‐specific shRNAs for 48 h and selected with puromycin (10 μg/ml) for another 72 h. The cells were then transfected with the indicated plasmids for 48 h. Cells were harvested for western blot analysis (G) and qRT‐PCR analysis (H). (I) 22Rv1 cells were infected with lentivirus vectors expressing control or HAT1‐specific shRNAs for 48 h and selected with puromycin (10 μg/ml) for another 72 h. The cells were then treated with or without JQ1 (3 μM) for 24 h and harvested for ChIP‐qPCR analysis. (J) A hypothetical model depicting the mechanism of HAT1 catalyzing histone H4 acetylation and recruiting BRD4 binding to the acetylated H4 and initiating AR transcription. Data were presented as the mean ± SD of three independent experiments. Error bars indicate SD. The *p* values were calculated using ANOVA (B and I) and Student's *t*‐tests (F and H). Spearman's correlation analysis was performed to determine the correlation between AR and BRD4, and the *p* value is shown as indicated (D). **p < 0.01, ***p < 0.001

Although we showed that HAT1 transcriptionally regulates the expression of AR, the underlying mechanism is poorly understood. There are multiple transcriptional factors, such as MYC, EZH2, TRIM24, KLF4, and BRD4, that can regulate AR transcription by binding to its promoter in tumor cells.^39^, ^40^, ^41^, ^42^, ^43^, ^44^ HAT1 can catalyze the H4K5 and H4K12 acetylation,^22^ which plays a key role in the process of BRD4 to bind to histone H4 and initiate transcription. As a result, we hypothesized whether BRD4 could serve as a mediator in the process of HAT1‐induced AR expression. To test our hypothesis, we utilized the constructed 22Rv1 cells with HAT1 knockdown and treated them with or without JQ1, a well‐known BRD4 inhibitor.^45^ We found that the HAT1 knockdown reduced AR expression (including AR‐FL and AR‐V7) as expected; however, this effect was impeded by JQ1 treatment (Figures 6A and 6B), in which KDM5C was set as a positive control to show the drug‐JQ1 working (Figure 6A).^46^ Notably, we assessed the existing ChIP‐seqence data reported by other groups and observed that BRD4 has a binding peak on the promoter of AR (Figure 6C). We further assessed the correlation between AR and BRD4 using the GEPIA webtool and noted that there is a strong positive correlation between the expression of AR and BRD4 (Spearman's correlation coefficient *R* = 0.59, *p* < 0.001, Figure 6D). Similarly, double knockdown of HAT1 and BRD4 did not further decrease AR expression compared with single HAT1 knockdown (Figure 6E), suggesting that HAT1 upregulates AR expression in a BRD4‐dependent manner. Consistently, when ectopically expressing HAT1, AR expression increased markedly, but this effect was almost entirely diminished after knocking down BRD4 in 22Rv1 cells (Figures 6G and 6H). In addition, the binding of BRD4 in 22Rv1 cells was confirmed by utilizing a ChIP‐qPCR assay. Further we observed that the binding of BRD4 on the AR promoter was decreased with HAT1 knockdown, or when cells were treated with JQ1, and such binding was not further decreased with combined HAT1 knockdown and JQ1 treatment (Figure 6I).

Collectively, these data demonstrate that HAT1 transcriptionally regulates AR expression via a BRD4‐mediated pathway and that HAT1 inhibition can re‐sensitize CRPC cells to treatment with ENZ by influencing both AR‐FL and AR‐Vs (Figure 6J).

## 4 DISCUSSION

Histone acetylation is a process of adding acetyl groups to histone proteins, and this process is crucial for relaxing of chromatin structure, thus allowing different genes to be transcribed and translated.^47^, ^48^ Hence, the study on histone acetyltransferases and deacetylases is to be particularly important. CBP/P300 is known histone acetyltransferases that have had more comprehensive studies in PCa.^49^, ^50^ For example, EP300/CREBBP inhibitors sensitize ENZ‐resistant PCa cells through inhibiting AR activity via affecting the MYC/ribosomal protein axis,^51^ implying that HATs play important roles in PCa progression and ENZ resistance. However, HAT1, a member of the histone acetyltransferase family, lacks exploration in PCa. In our study, we performed a series of experiments to demonstrate that HAT1 is highly expressed in PCa and positively correlated with PCa progression. Knockdown of HAT1 significantly impeded the PCa growth, while over‐expressing HAT1 could accelerate PCa proliferation.

As a well‐acknowledged malignant factor that drives PCa initiation and progression,^9^, ^52^ AR exerts its oncogenic function mainly by transcriptionally binding on the promoter or enhancer of its target genes and stimulating their expression, further stimulating the AR signaling pathway.^53^ Evidence has shown that targeting AR could effectively curb the deterioration of PCa.^54^, ^55^, ^56^, ^57^ As a result, ADT is always the initial and mainstay therapy for PCa treatment. However, although effective at the beginning, the disease in almost all patients relapses into CRPC less than 2 years after ADT treatment.^58^, ^59^ Hence, a comprehensive understanding of the AR signaling regulation in PCa is worth exploration. In this study, we observed that HAT1 upregulated AR expression at the transcriptional level. Conversely, with knockdown of HAT1, the mRNA expression of *KLK3*, *TMPRSS2*, and *NKX3.1* decreased. Alternatively, when HAT1 was overexpressed in cell lines, AR expression was increased at both the protein and mRNA levels. Furthermore, in human PCa samples, AR expression is also positively correlated with HAT1. Together, these data strongly suggested that HAT1 upregulated AR expression, which may be one of reasons that PCa relapses into CRPC after ADT treatment.

Among the ADT chemical drugs, ENZ is one of the most used next‐generation androgen signaling inhibitors for the treatment of CRPC. Unfortunately, PCa cells become resistant to this treatment method. It has been reported PCa cells develop AR‐Vs,^14^ some of which lack the LBD binding domain that binds to ENZ, leading to ENZ resistance.^60^ Due to emerging these AR‐Vs, PCa patients often failed ENZ treatment, and CRPC progression occurs.^16^ Among these AR‐Vs, AR‐V7 is the most notorious one that closely relates with ENZ resistance.^16^, ^17^ Therefore, an effective therapeutic to reverse ENZ resistance is needed. There are already multiple ways to target AR‐V7 for CRPC treatment, but no promising results have been demonstrated.^61^ Our results showed that HAT1 could be a potential target for CRPC treatment due to its positive regulation of AR‐FL, especially AR‐V7. We further demonstrated that such regulation occurred at the mRNA level. Given that AR‐V7 has been restrained, we tested the influence of HAT1 inhibition on ENZ efficacy using 22Rv1 cells which are ideal model because they express high levels of AR‐FL and AR‐V7. These results excitingly showed that HAT1 knockdown could re‐sensitize 22Rv1 cells to ENZ treatment. This effect may result from the decreased AR expression when HAT1 is knocked down, and noticeably, HAT1 knockdown had a more distinct impact on AR‐V7 as we observed in Figure 3B, which may contribute to the ENZ efficacy.

As to how HAT1 regulates AR expression, there are multiple transcriptional factors, such as MYC, EZH2, TRIM24, KLF4, and BRD4, regulate AR transcription by binding to its promoter in tumor cells.^39^, ^40^, ^41^, ^42^, ^43^, ^44^ HAT1 can catalyze the H4K5 and H4K12 acetylation^22^ that is crucial for BRD4 to bind to histone H4 and initiate transcription. In this study, we hypothesized whether BRD4 could serve as a mediator in the process of HAT1‐induced AR expression. As a result, we observed that BRD4 has a binding peak on the promoter of AR and noted that there is a positive correlation between the expression of AR and BRD4 (Figure 6). Further, we found that the binding of BRD4 on the AR promoter was decreased when HAT1 was knocked down or when cells were treated with JQ1, and such binding did not further decrease with combined HAT1 knockdown and JQ1 treatment, indicating that BRD4 could serve as a mediator in the process of HAT1‐induced AR expression. However, the detailed mechanism underlying HAT1 transcriptionally regulating the expression of AR still needs further investigation.

Because there is no specific HAT1 inhibitor, we used a recently reported HAT1 repressor, ascorbate, an essential dietary substance with pro‐oxidant property that modulates AR‐mediated signaling in Los Angeles PC (LAPC)‐4 cells,^62^ to target HAT1 and found that ascorbate successfully decreased AR expression (including AR‐V7 expression) by influencing HAT1 in PCa (Figures 3H–3K), which further indicates that HAT1 plays an important effect in regulating the expression of AR and CRPC resistance to ADT treatment and implies that developing a small molecular HAT1‐specific inhibitor may be of great use in cancer treatments.

Although HAT1 can promote PCa and CRPC progression through upregulating AR (including AR‐V7) expression via a BRD4‐mediated pathway and knockdown of HAT1 partially re‐sensitizes CRPC cells to ENZ treatment, there are still some problems to be further studied. Given that HAT1 upregulates AR expression via a BRD4‐mediated pathway, the underlying connection mechanisms between the BRD4 and the HAT1 affection on AR have not been thoroughly investigated. In addition, although ascorbate efficiently decreases AR expression by influencing HAT1 in PCa cells *in vitro* experiments, this effect of ascorbate on AR in *in vivo* experiments warrants further probe to prove its potential applications in modulating ENZ sensitivity.

## 5 CONCLUSIONS

In summary, HAT1, a previously unrecognized regulator of AR, transcriptionally upregulates AR expression in PCa cells through a BRD4‐mediated pathway, and inhibition of HAT1 re‐sensitizes CRPC cells to ENZ treatment, suggesting that HAT1 serves as a critical oncoprotein and an ideal target for the treatment of ENZ‐resistant CRPC patients.

## CONFLICT OF INTEREST

The authors declare no conflict of interest.

## AUTHOR CONTRIBUTIONS

Zhe Hong and Zhendong Xiang conceived and designed the experiments. Zhe Hong, Zhendong Xiang, Pan Zhang, Xinan Wang, Chengdang Xu, and Qiang Wu performed the *in vitro* and *in vivo* experiments and analyzed the results. Zhe Hong and Zhendong Xiang wrote the manuscript. Zongyuan Hong, Guowei Shi, and Denglong Wu revised and edited the manuscript. All authors read and approved the final manuscript.

## Supporting information

SUPPORTING INFORMATION

SUPPORTING INFORMATION

SUPPORTING INFORMATION

SUPPORTING INFORMATION

## Data Availability

The data used or analyzed during this study are included in this article and available from the corresponding author.
