# Supplementary figures and images for: Histone acetyltransferase 1 upregulates androgen receptor expression to modulate CRPC cell resistance to enzalutamide
Source: Clin Transl Med. 2021 Jul 22;11(7):e495. doi: 10.1002/ctm2.495 (PMC8299045; doi:10.1002/ctm2.495)

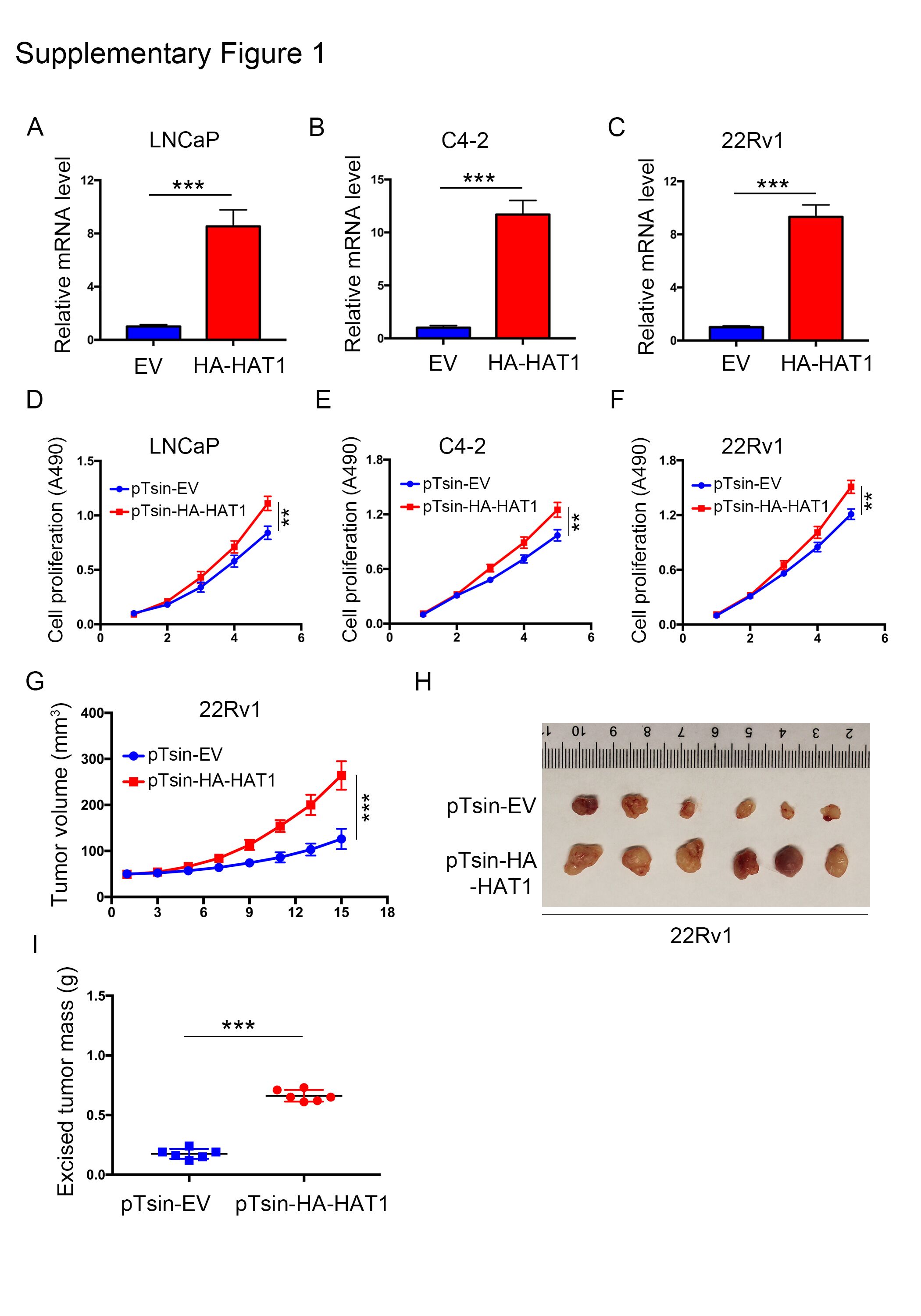

Supplement: Supplementary file 1 — SUPPORTING INFORMATION [file CTM2-11-e495-s003.jpg]

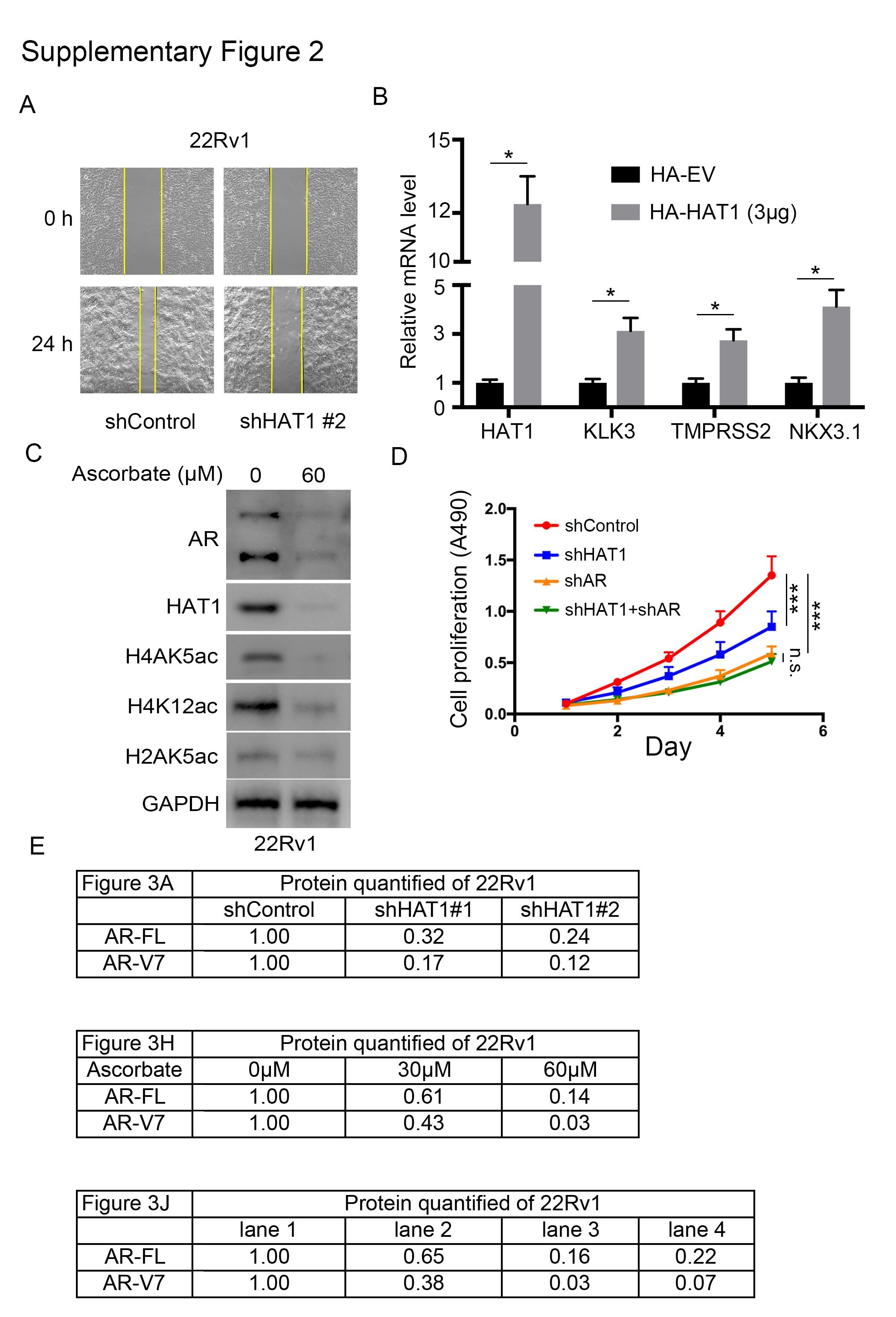

Supplement: Supplementary file 2 — SUPPORTING INFORMATION [file CTM2-11-e495-s001.jpg]

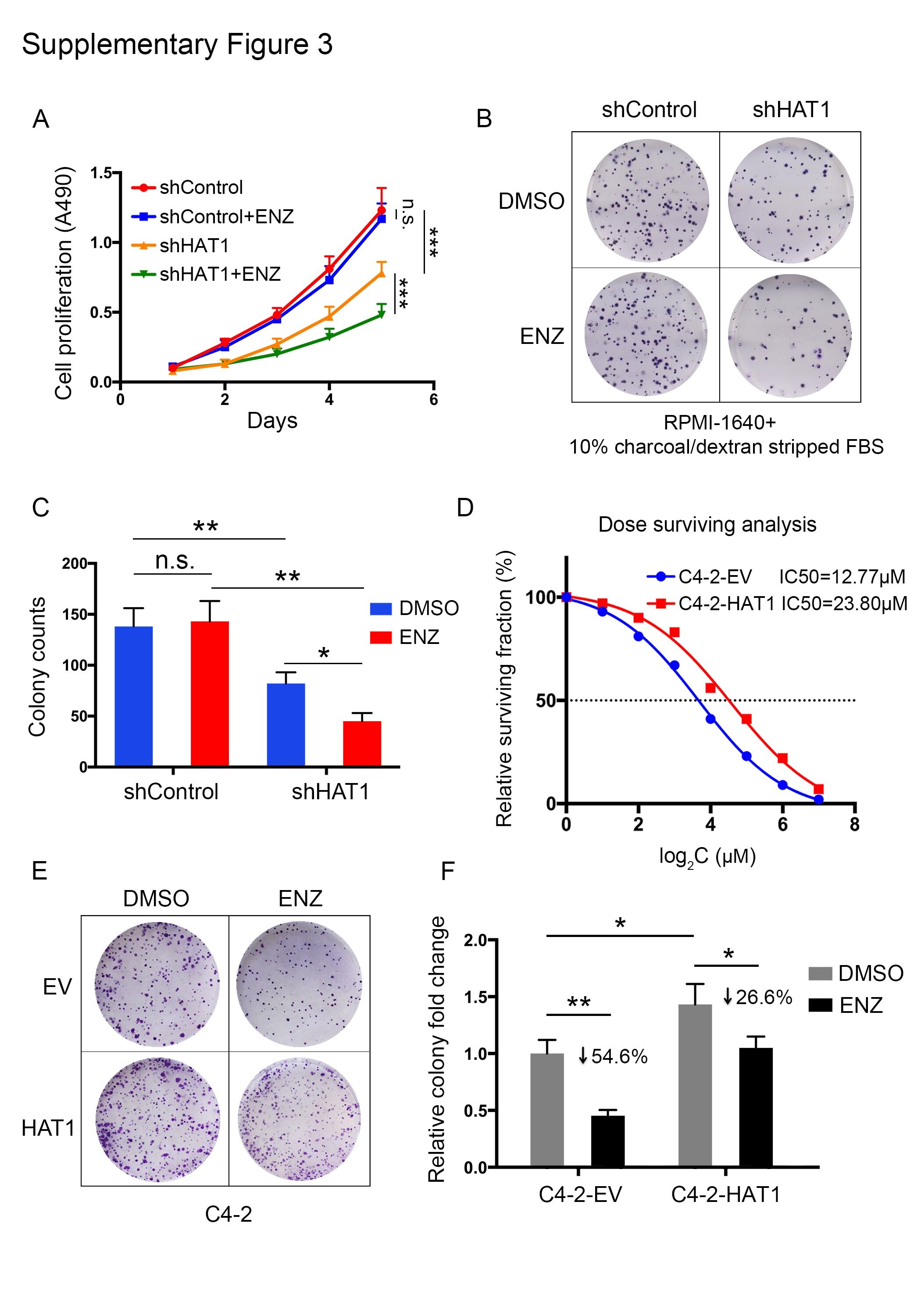

Supplement: Supplementary file 3 — SUPPORTING INFORMATION [file CTM2-11-e495-s004.jpg]

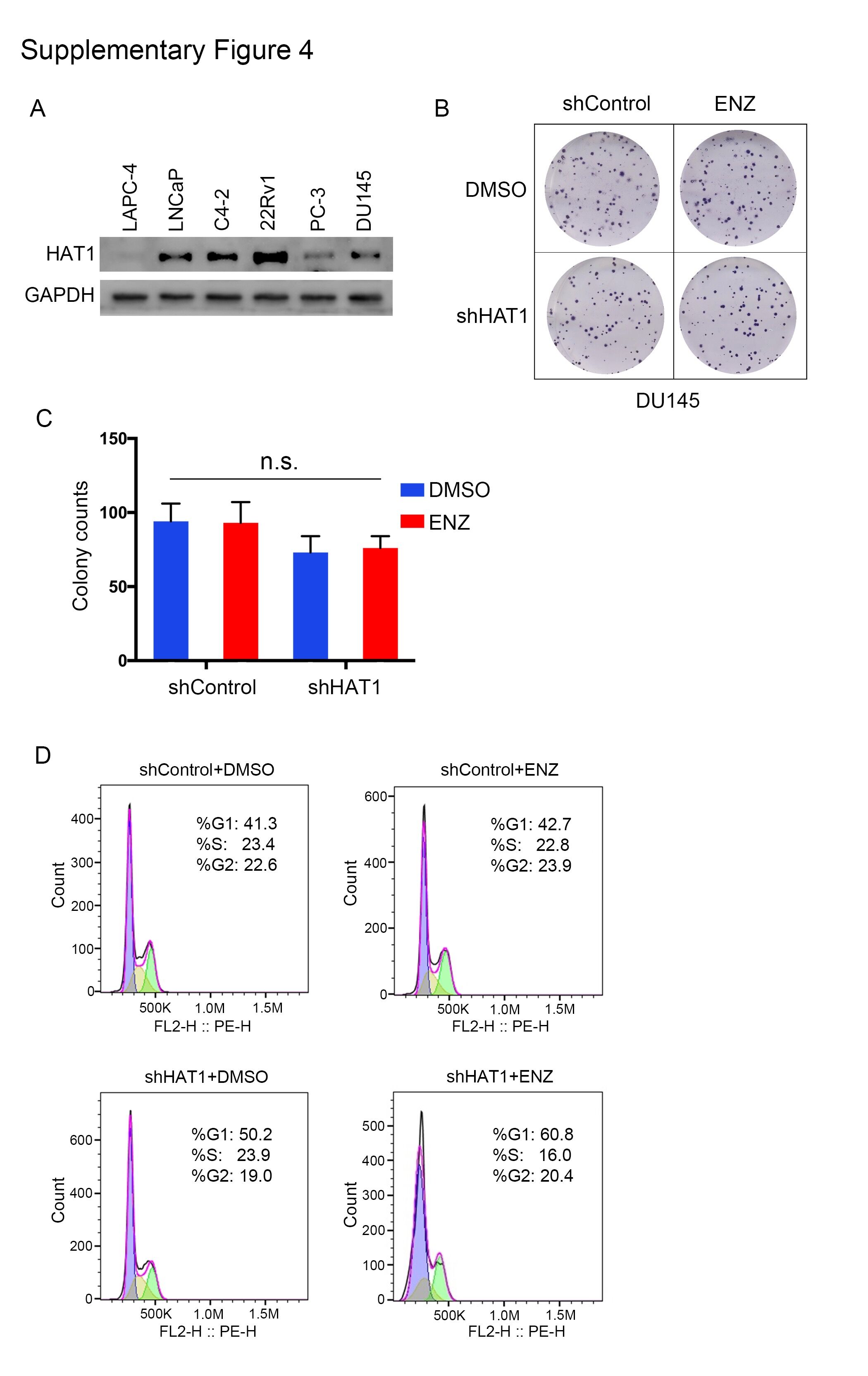

Supplement: Supplementary file 4 — SUPPORTING INFORMATION [file CTM2-11-e495-s002.jpg]
